# Detection of *SRSF2-*P95 Mutation by High-Resolution Melting Curve Analysis and Its Effect on Prognosis in Myelodysplastic Syndrome

**DOI:** 10.1371/journal.pone.0115693

**Published:** 2014-12-26

**Authors:** Jiang Lin, Jing Yang, Xiang-mei Wen, Lei Yang, Zhao-qun Deng, Zhen Qian, Ji-chun Ma, Hong Guo, Ying-ying Zhang, Wei Qian, Jun Qian

**Affiliations:** 1 Laboratory Center, Affiliated People's Hospital of Jiangsu University, Zhenjiang, Jiangsu, People's Republic of China; 2 Department of Hematology, Affiliated People's Hospital of Jiangsu University, Zhenjiang, Jiangsu, People's Republic of China; University of New England, Australia

## Abstract

Hotspot mutations of serine/arginine-rich splicing factor 2 (*SRSF2*) gene have been identified in a proportion of hematologic malignancies including myelodysplastic syndrome (MDS). The aim of the present study was to develop a new approach to screen *SRSF2* mutation and analyze the clinical relevance of *SRSF2* mutations in Chinese MDS. A protocol based on high-resolution melting analysis (HRMA) was established to screen *SRSF2-*P95 mutation in 108 MDS patients and was compared with Sanger sequencing. The clinical relevance of *SRSF2* mutations was further evaluated. HRMA identified five (4.6%) cases with *SRSF2* mutation, completely validated by Sanger sequencing without false positive or negative results. The sensitivities of HRMA and Sanger sequencing were 10% and 25% for the detection of *SRSF2*-P95H mutation, respectively, against the background of wild-type DNA. Patients with *SRSF2* mutation had shorter overall survival time than those with wild-type *SRSF2* in both the whole cohort of cases and those with normal karyotype (*P* = 0.069 and 0.023, respectively). Multivariate analysis confirmed *SRSF2* mutation as an independent risk factor in both patient populations. We established a fast, high-throughput, and inexpensive HRMA-based method to screen *SRSF2* mutation, which could be used in clinical diagnostic laboratories. *SRSF2* mutations were significantly associated with mortality rate in the MDS affected Chinese.

## Introduction

During the process of gene expression in eukaryotic cells, pre-mRNA undergoes a fundamental process of removal of introns and ligation of exons to form mature mRNA transcripts, i.e., splicing [1], [2]. Alternative pre-mRNA splicing, occurring in the majority of human genes, produces a large number of alternatively spliced mRNA isoforms with various biological properties and contributes to functional diversity and complexity in physiologic conditions [3], [4]. A growing number of studies demonstrate that aberrant alternative splicing is involved in the pathogenesis of cancers including hematologic malignancies [5]–[7]. Mutations of several genes crucial for the splicing process have been found to contribute to tumorigenesis [5], [8].

Serine/arginine-rich splicing factor 2 (SRSF2) belongs to the family of serine/arginine-rich (SR) proteins that play essential roles in the control of constitutive and alternative pre-mRNA splicing [9]–[11]. A heterozygous hotspot mutation in *SRSF2* gene has been identified in hematologic malignancies with varying frequencies, including myelodysplastic syndrome (MDS) [12]–[16], acute myeloid leukemia (AML) [12], chronic myelomonocytic leukemia (CMML) [17], [18], primary myelofibrosis (PMF) [19], [20], and systematic mastocytosis [21]. *SRSF2* mutation appears to predict adverse prognosis in MDS and PMF [14], [16], [20], [22]. Therefore, the detection of *SRSF2* mutation may be helpful to the diagnosis and risk classification in hematologic malignancies.

Currently, two methods, including direct DNA sequencing and PCR-single strain conformation polymorphism (SSCP), have been applied to detect *SRSF2* mutation [23]. However, these two methods are not suitable for high-throughput screening of mutations as they are costly and labor intensive. More convenient method should be developed in the field of molecular diagnostics and risk stratification of hematologic malignancies for the detection of gene mutations that occur in low incidence. In this study, we established a fast and efficient approach to screen *SRSF2* mutation using high-resolution melting analysis (HRMA) [24] and analyzed the clinical significance of *SRSF2* mutations in MDS.

## Materials and Methods

### Patients' samples

This study was approved by the Ethics Committee Board of Affiliated People's Hospital of Jiangsu University. The bone marrow (BM) samples from 108 patients with de novo MDS were collected at the time of initial diagnosis after the informed consent was written. The diagnosis and classification of MDS were based on the French-American-British (FAB) and the World Health Organization (WHO) classification [25], [26]. Risk groups were classified according to International Prognosis Scoring System (IPSS) [27]. Clinical characteristics of all patients were listed in Table 1.

**Table 1:** Distribution of *SRSF2* mutations in MDS patients.

	Total	*SRSF2* mutation		*P*
	n = 109	_+_ (n = 5)	− (n = 103)	
Sex, male/female	64/45	5/0	59/44	1.000
Median age, years (range)	60 (20–86)	68 (60–77)	58 (20–86)	0.176
Median WBC,×10^9^/L (range)	2.7 (0.6–82.4)	1.9 (1.2–26.6)	2.7(0.6–82.4)	0.288
Median hemoglobin, g/L(range)	63 (26–128)	71 (62–88)	62 (26–128)	0.305
Median platelets,×19^9^/L(range)	62 (1–1176)	60 (43–323)	63 (1–1176)	0.330
WHO, no.				0.766
RA	10	0	10	
RARS	0	0	1	
RCMD	36	2	34	
RCMD-RS	8	0	8	
RAEB-1	23	2	21	
RAEB-2	25	1	24	
Del (5q)	5	0	5	
MDS-U	1	0	1	
Karyotype classification				0.747
Favorable	81	4	75	
Intermediate	17	1	16	
Poor	8	0	8	
No data	4	0	4	
IPSS				0.577
Low	10	0	10	
Int-1	62	4	58	
Int-2	20	1	19	
High	12	0	12	
^ ^No data	4	0	4	

WBC, white blood cell count; WHO, World Health Organization; RA, refractory anemia; RARS, refractory anemia with ringed sideroblasts; RCMD, refractory cytopenia with multilineage dysplasia; RCMD-RS, refractory cytopenia with multilineage dysplasia with ringed sideroblasts; RAEB, refractory anemia with excess of blasts; del(5q), MDS with isolated del(5q); MDS-U, myelodysplastic syndrome unclassifiable; IPSS, International Prognostic Scoring System.

### DNA extraction

BM mononuclear cells were separated by density-gradient centrifugation using Ficoll. DNA was extracted using the Genomic DNA Purification Kit (Gentra, Minneapolis, MN, USA) according to the manufacturer's instructions.

### PCR and HRMA

Primers for PCR-HRMA to screen *SRSF2-*P95 hotspot mutation were designed with LightScanner primer design software v1.0 (Idaho, Salt Lake City, Utah). The sequences of primer set were 5′- TGCAAATGGCGCGCTAC -3′ (forward) and 5′- GGCGGCTGTGGTGTGAG -3′ (reverse) with an expected PCR product of 48bp.

PCR was carried out in 25-µl volume in the presence of 1× PCR buffer (Takara, Tokyo, Japan), 0.2 mmol/l of each dNTP, 0.5 µmol/l of both forward and reverse primers, 0.8 µmol/l of internal oligonucleotide calibrators [28], 1×LCgreen Plus (Idaho, Salt Lake City, Utah, USA), 0.75 U hot start DNA polymerase (Takara, Tokyo, Japan), and 50 ng genomic DNA. PCR reactions were performed on a 7300 Thermo cycler (Applied Biosystems, Foster City, CA, USA). The thermal cycling was 10 minutes at 98°C, followed by 40 cycles of 98°C for 10 seconds, 63°C for 30 seconds, and 72°C for 30 seconds, followed by a final 7 min extension step at 72°C.

After PCR, 96-plate was transferred to the LightScanner (Idaho, Salt Lake City, Utah, USA) for HRMA. Plates were heated from 55°C up to 95°C with a ramp rate of 0.10°C/s. The melting curve analysis was carried out by the LightScanner software package with CALL-IT module (Idaho, Salt Lake City, Utah, USA). Melting profiles were calibrated by internal oligonucleotide control, and then normalized, grouped and displayed as fluorescence-versus-temperature plots or subtractive difference plots (-df/dt vs T).

### PCR and Sanger sequencing

In order to validate the results of HRMA, another primer pair was designed with Primer Premier 5.0 (PremierBiosoft, Palo Alto, CA, USA) to amplify a larger PCR product (212 bp). The primer sequences were 5′- TTCGCCTTCGTTCGCTTTCA -3′ (forward) and 5′- CCCCTCAGCCCCGTTTACC -3′ (reverse). PCR reaction system was similar to PCR-HRMA except that no LCgreen Plus was included in the reaction and thermal cycling was same as PCR-HRMA cycles with a modification of the annealing temperature to 60°C. PCR products were directly sequenced on both strands using an ABI 3730 automatic sequencer.

### Statistics

Statistical analysis was performed using the SPSS 17.0 software package (SPSS, Chicago, IL, USA). The difference of categorical variables was compared by Pearson Chi-square analysis or Fisher exact test. The difference of continuous variables was compared by Mann-Whitney's U test. Survival was analyzed according to the Kaplan–Meier method. The Cox regression backward stepwise likelihood ratio was also used for multivariate analysis. For all analyses, the *P* values were two-tailed, and less than 0.05 was considered statistically significant.

## Results

### Identification of *SRSF2*-P95 mutation with HRMA

**Figure 1:**
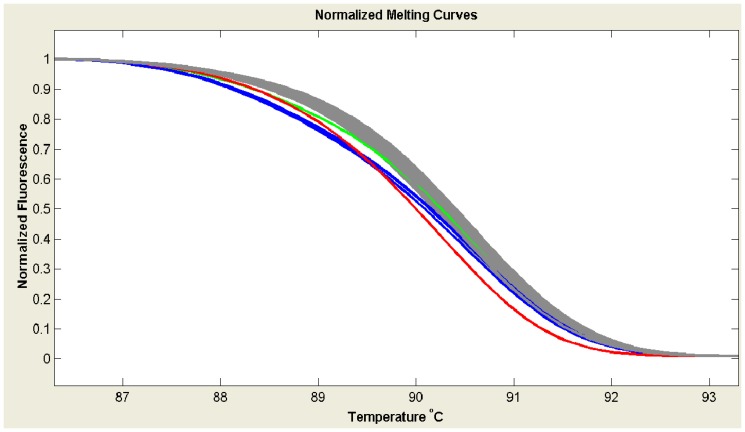
*SRSF2* P95 mutation detected by PCR-HRMA in MDS patients. Grey lines represented wild-type *SRSF2*; green and blue lines were validated as P95H mutations by Sanger sequencing; red line was validated as P95L mutation.

**Figure 2:**
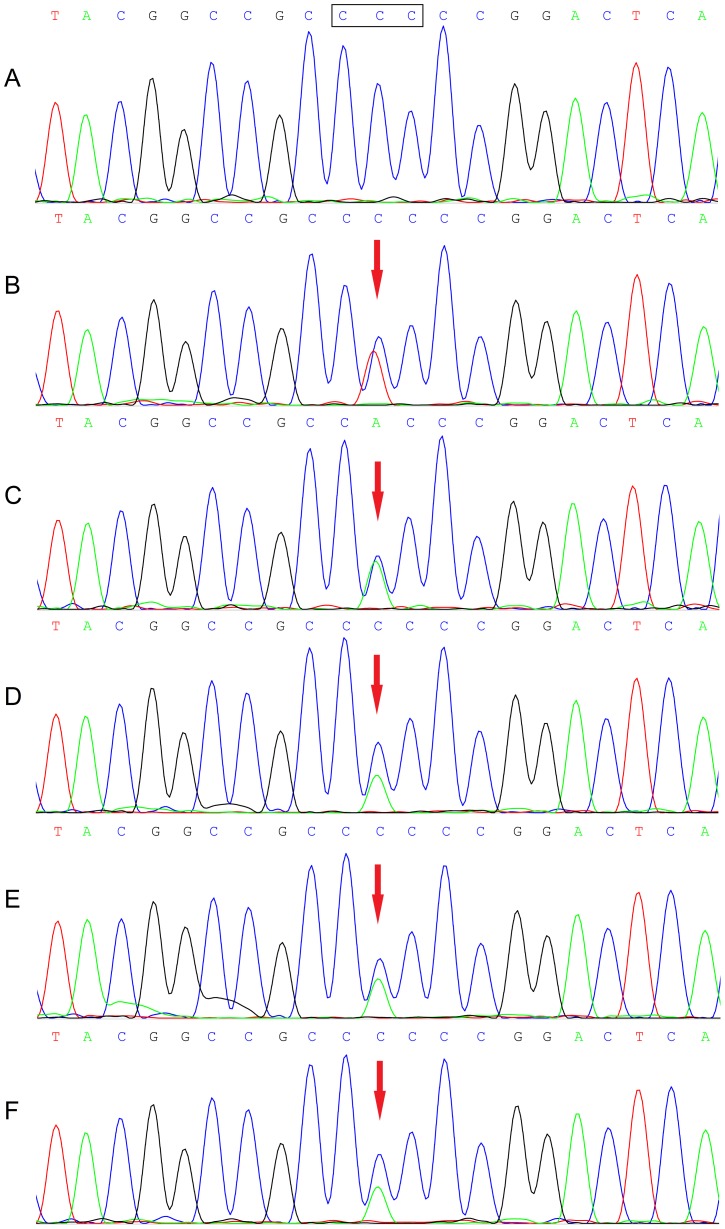
Sequence chromatogram of *SRSF2*-P95 mutation. A: wild-type *SRSF2*; B: P95L mutation (c.284C>T); C-F: P95H mutation (c.284C>A). The rectangle denoted the ninety-fifth codon of *SRSF2* gene. The mutated nucleotide was indicated by a red arrow.

We firstly screened *SRSF2-*P95 mutations in our cohort of MDS patients using HRMA and identified five (4.6%) cases with aberrant melting curves (Fig. 1) that were suspected with mutations. All of 108 MDS samples were then direct DNA sequenced. Sanger sequencing completely validated the results of HRMA without false positive or negative results. Among the five mutations, one was heterozygous P95L (c.284C>T) and four were heterozygous P95H (c.284C>A) (Fig. 2).

**Figure 3:**
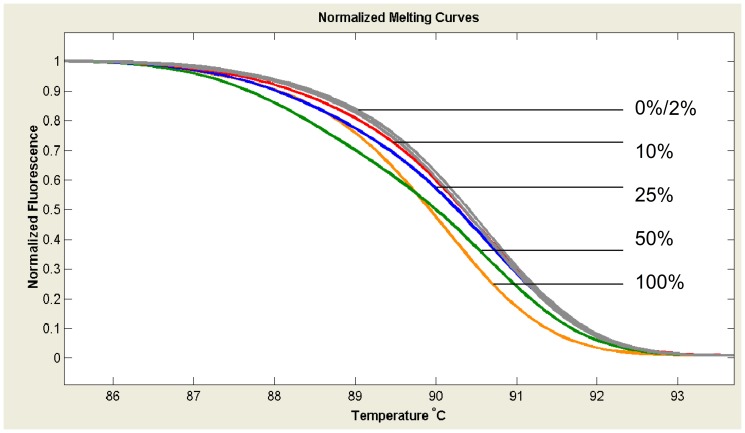
Sensitivity of HRMA in detecting *SRSF2*-P95H mutation. The numbers represented the concentration of P95H plasmid diluted with wild-type plasmid. The maximal sensitivity was 10%.

**Figure 4:**
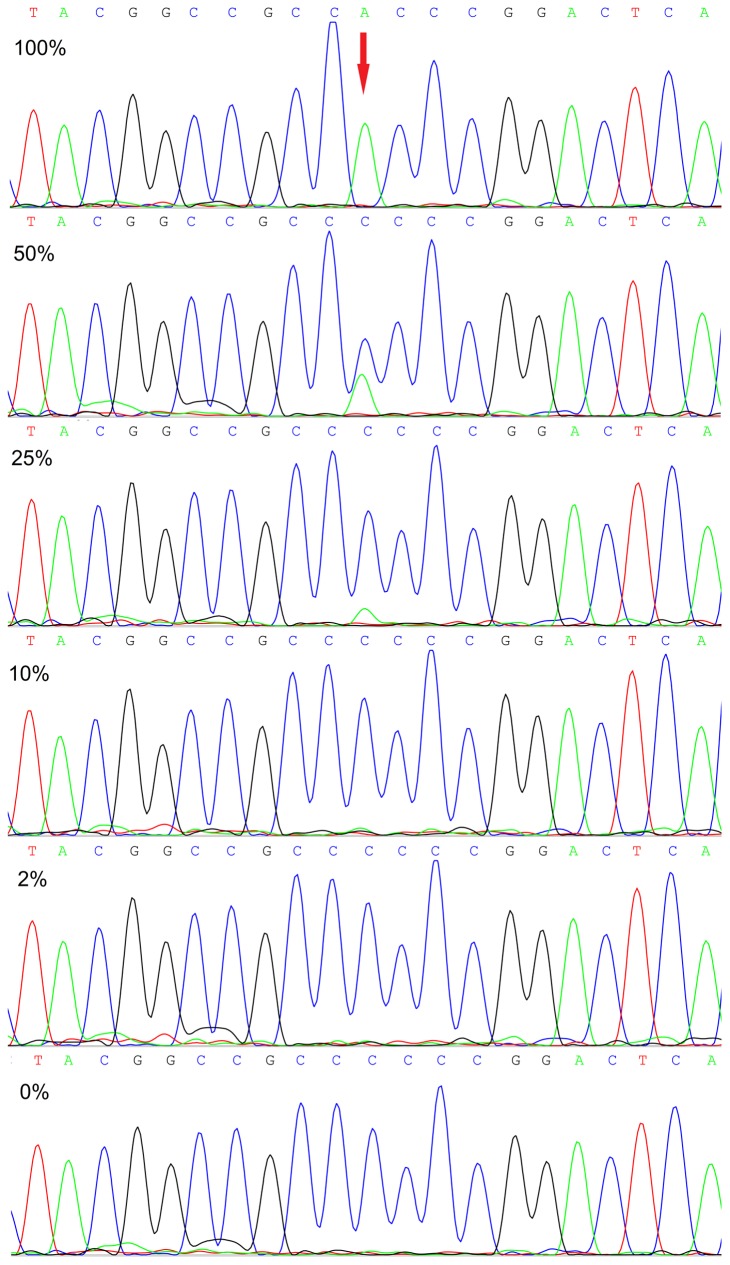
Sensitivity of Sanger sequencing in detecting *SRSF2*-P95H mutation. The numbers represented the concentration of P95H plasmid diluted with wild-type plasmid. The maximal sensitivity was 25%.

To further determine the sensitivity of HRMA in the identification of *SRSF2*-P95 mutations, purified plasmid DNA cloned with *SRSF2*-P95H mutant or wild type was generated from one AML patient identified with heterozygous P95H mutation by sequencing. We evaluated the sensitivity of HRMA by analyzing plasmid DNA with different concentrations of P95H mutant serially diluted by wild type (0% mutant, 1% mutant, 2% mutant, 10% mutant, 25% mutant, 50% mutant, and 100% mutant). The sensitivity test was carried out in duplicates to ensure the reproducibility of the HRMA. *SRSF2*-P95H mutation could be easily distinguished with the maximal sensitivity of 10% mutated sample in a background of wild-type DNA (Fig. 3). However, mutated P95H was identified at the maximal sensitivity of 25% by direct DNA sequencing (Fig. 4).

### Clinical implications of *SRSF2* P95H mutations in MDS patients

There were no difference in age, blood parameters, WHO subtypes, IPSS subgroups and karyotype classification between cases with and without mutations (*P*>0.05, Table 1). Although all five *SRSF2* mutations were observed in male patients, the association of *SRSF2* mutation with gender could not be found (Table 1). The main clinical features of five patients with *SRSF2* mutations were listed in Table 2.

**Table 2:** The clinical and hematopoietic parameters of 5 patients with *SRSF2* mutations.

ID	Sex/Age (years)	Diagnosis	WBC (×10^9^/L)	Hemoglobin (g/L)	Platelet (×10^9^/L)	Karyotype	Survival time (months)	*SRSF2* mutation
1	M/76	RCMD	1.2	62	43	i(17q)	10	P95H
2	M/60	RAEB-1	1.9	70	60	N	26	P95H
3	M/68	RAEB-1	1.7	71	323	N	16	P95H
4	M/77	RAEB-2	26.6	88	234	N	1	P95H
5	M/62	RCMD	2.5	74	49	N	9	P95R

M, male; N, normal.

**Figure 5:**
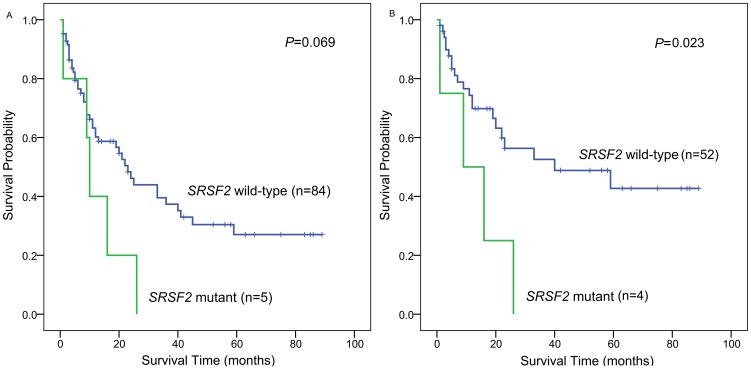
Overall survival in MDS patients according to Kaplan-Meier analysis. A: all patients; B: cytogenetically normal patients.

The follow-up data were obtained for 89 MDS patients. The median follow-up duration of the patients was 11 months (range, 1–89 months). The overall survival (OS) of MDS patients with *SRSF2* mutation (median 10 months, 95% confidence interval 7.8–12.1 months) was shorter than those without mutation (median 23 months, 95% confidence interval 17.1–28.9 months) (*P* = 0.069, Fig. 5A). Cox proportional hazard regression model was carried out including five parameters including sex, age, abnormal neutrophils count (ANC), IPSS grouping, and *SRSF2* mutation. The results showed that *SRSF2* mutation and IPSS grouping were independent prognostic factors (Table 3). Furthermore, in MDS patients with normal karyotype, *SRSF2* mutation was also found to predict the adverse outcome by Kaplan-Meier, univariate and multivariate Cox analyses (Fig. 5B, Table 4).

**Table 3:** Univariate and multivariate analyses for OS in MDS patients.

	OS univariate analysis			OS multivariate analysis		
	HR	95% CI	*P*	HR*	95% CI	*P*
Sex (male vs female)	1.995	1.080–3.685	0.027	1.911	0.922–3.962	0.082
Age (≥60 vs <60 years)	2.571	1.404–4.717	0.002	1.961	0.923–3.953	0.060
ANC (<1.8 vs>1.8 ×10^9^/l)	0.829	0.470–1.463	0.517	0.802	0.393–1.636	0.544
IPSS (Int-2/High vs Low/Int-1)	2.033	1.082–3.821	0.027	4.947	2.296–10.657	<0.001
*SRSF2* mutation (yes vs no)	2.342	0.918–5.988	0.075	3.315	1.009–9.709	0.048

OS, overall survival; CI, confidential interval; HR, hazard ratio; *HR>1 indicates an increased risk of an event for the first category listed.

**Table 4:** Univariate and multivariate analyses for OS in MDS patients with normal karyotype.

	OS univariate analysis			OS multivariate analysis		
	HR	95% CI	*P*	HR*	95% CI	*P*
Sex (male vs female)	2.075	0.863–4.987	0.103	2.000	0.790–5.065	0.144
Age (≥60 vs <60 years)	4.161	1.680–10.306	0.002	3.533	1.375–9.075	0.009
ANC (<1.8 vs>1.8×10^9^/l)	0.860	0.389–1.900	0.710	0.528	0.211–1.321	0.172
IPSS (Int-2/High vs Low/Int-1)	2.601	1.065–6.354	0.036	2.535	0.968–6.641	0.058
*SRSF2* mutation (yes vs no)	3.258	1.100–9.647	0.033	4.257	1.316–13.767	0.016

OS, overall survival; CI, confidential interval; HR, hazard ratio; *HR>1 indicates an increased risk of an event for the first category listed.

## Discussion

In this study, we first established a fast, reliable, high-throughput, and inexpensive HRMA-based method to screen patients for *SRSF2* mutations. After DNA isolation and PCR, HRMA was rapid and convenient, using a closed single well system, without the requirement of any post-PCR handling of solutions. Afterwards, only samples positive in HRMA were required to be sequenced to identify the base pair changes, thereby significantly reduced time-consuming sequencing work with significantly lower costs. The application of HRMA method is very useful in the field of molecular diagnostics and risk stratification of hematologic malignancies for the detection of gene mutations that occur in low frequencies. We identified 4.6% of *SRSF2* mutations, lower than reported previously (11.1%–15%) [12]–[16], [23], [29], [30]. However, the low incidence of *SRSF2* mutations in this study was not due to the detection limit caused by HRMA method itself: firstly, HRMA was more sensitive than Sanger sequencing; secondly, all samples were confirmed by Sanger sequencing which found no other mutations except for those identified by HRMA. Yet, it should be noted that HRMA remains a limitation for identification of low proportion (<10%) of *SRSF2* mutations in the sample.

We next analyzed the clinical relevance of *SRSF2* mutations in our cohort of MDS patients. Although all five *SRSF2* mutations were found in male patients and *SRSF2* mutated patients were older than those with wild-type *SRSF2*, the association of *SRSF2* mutation with gender and age was not observed in this study. Wu et al (2012) reported that *SRSF2* mutation was closely associated with male sex and older age in the Taiwanese population [16]. Thol et al (2012) also observed the obvious trend that *SRSF2* mutation was predominant in male patients [14]. Unlike mutations in *SF3B1* gene [31], *SRSF2* mutations were not associated with the subtypes with ring sideroblasts. Instead, *SRSF2* mutation occurred in all different subtypes of MDS and was not associated with a specific IPSS risk as reported previously [13], [14], [16].

*SRSF2* mutations predicted a negative prognostic impact in our study. Patients with this mutation showed an obvious trend towards inferior overall survival according to univariate analysis in the whole patient cohort with follow-up data, while multivariate analysis revealed that *SRSF2* mutation was an independent adverse prognostic factor in MDS. Furthermore, Kaplan-Meier, univariate and multivariate Cox analyses also disclosed the unfavorable impact of *SRSF2* mutation on outcome in MDS patients with normal karyotype. However, the therapy regimen, which can alter the natural history of the diseases, was not uniform in this study. Moreover, the presence of other mutations (such as *TP53*, *EZH2*, *RUNX1*, or *ASXL1*) which could also alter survival was not investigated in this study. These may affect the accurate assessment of the influence of *SRSF2* mutation on the survival. The impact of *SRSF2* mutation on prognosis remains controversial currently. Two recent studies have suggested that *SRSF2* mutation is prognostically unfavorable [14], [16], while others have not so [13], [21], [23], [30]. Obviously, prospective studies are needed to determine whether *SRSF2* mutation could be used as a potential marker for risk stratification in MDS.

In conclusion, we established a fast, high-throughput, and inexpensive HRMA-based method to screen *SRSF2* mutation, which could be used in clinical diagnostic laboratories. We identified *SRSF2* mutations in 4.6% of Chinese MDS patients and found a significant association of *SRSF2* mutation and unfavorable outcome of survival of the patients.
